# The Effects of *Sorbus aucuparia* L. Fruit Extracts on Oxidative/Nitrative Modifications of Human Fibrinogen, Impact on Enzymatic Properties of Thrombin, and Hyaluronidase Activity In Vitro

**DOI:** 10.3390/antiox10122009

**Published:** 2021-12-18

**Authors:** Magdalena Rutkowska, Joanna Kolodziejczyk-Czepas, Monika Anna Olszewska

**Affiliations:** 1 Department of Pharmacognosy, Faculty of Pharmacy, Medical University of Lodz, 1 Muszynskiego St., 90-151 Lodz, Poland; monika.olszewska@umed.lodz.pl; 2 Department of General Biochemistry, Faculty of Biology and Environmental Protection, University of Lodz, 141/143 Pomorska St., 90-236 Lodz, Poland; joanna.kolodziejczyk@biol.uni.lodz.pl

**Keywords:** *Sorbus aucuparia*, plasma haemostasis, fibrinogen, thrombin enzymatic activity, hyaluronidase, blood clotting times

## Abstract

*Sorbus aucuparia* L. fruits (rowanberries) are food products with acknowledged nutritional value, high phenolic content, and traditional application in diabetes. In this study, the effects of rowanberry extracts (phytochemically standardised, i.a., by LC-MS/MS) on some aspects of plasma haemostasis and vascular conditions were evaluated in vitro as possible mechanisms connected with cardiovascular complications of diabetes. The analyses of structural modifications of human fibrinogen under oxidative stress conditions (C-ELISA, SDS-PAGE and Western blot) revealed that the extracts (at a concentration of 1–5 µg/mL) considerably reduced the nitration of tyrosine residues and formation of high-molecular-weight aggregates. Moreover, they inhibited the enzymatic activity of thrombin (both amidolytic and proteolytic). Additionally, some promising outcomes might be expected regarding endothelial functions from the extracts ability to inhibit hyaluronidase. Parallel experiments on model polyphenols and correlation studies formed the basis for determining the contribution of different compounds, including hydroxycinnamic acid derivatives, flavonols, and low- or high-molecular-weight flavan-3-ols derivatives (proanthocyanidins), to the observed effects. The possible synergistic activity of individual constituents was also noticed. These results broaden the knowledge on the biological activity of rowanberries, partly confirming their health-promoting properties, and indicating that their functional applications might be promising.

## 1. Introduction

*Sorbus aucuparia* L. is a small, deciduous tree or shrub, widely occurring in Europe and producing fruits (rowanberries, rowan fruits) with well-documented nutritional value that are consumed as marmalades, jams, jellies, wines, and confectionery [1,2]. Their health-promoting properties have also been noticed, and they are used as a traditional herbal remedy for several complaints, especially for diabetes and diabetes-related cardiovascular complications [1,3]. Indeed, according to previous research, rowanberries exhibit inhibitory activity towards α-amylase and α-glucosidase, i.e., enzymes involved in the digestion of carbohydrates [4,5], and they turn out to be effective reducers of the sugar level in a mouse model of diabetes [6]. Moreover, rowanberry extracts were proved to inhibit the formation of advanced glycation end products [7], which are considered to be responsible for macro- and microvascular complications of diabetes [8]. Taking into account that glycation may both directly impair the function of various proteins and entail further damages to proteins and other biomolecules by increasing the production of reactive oxygen species (ROS) [8], the significant antioxidant activity of rowanberries has also been pointed out as an essential component of their health-promoting properties [7]. Among the previously observed effects of *S. aucuparia* fruit extracts on redox homeostasis were the ability to scavenge in vivo-generated ROS, increase the total antioxidant capacity of human plasma, and protect plasma proteins and lipids against oxidative and nitrative damage [7]. The occurrence of a rich fraction of various polyphenolic compounds, including proanthocyanidins, pseudodepsides of caffeic and ferulic acids, and flavonols, was indicated in rowanberries as co-responsible for their antioxidant and antiglycation properties and the ability to inhibit the digestion of carbohydrates [4,5,7]. So far, the obtained results seem to confirm the beneficial biological effects of rowan fruits and encourage more detailed studies on the bioactivity mechanisms of the tested plant material that might contribute to its functional applications.

Haemostasis involves a complex network of interactions between blood vessel walls, platelets, plasma coagulation cascade, and fibrinolysis proteins, responsible for maintaining the balance between procoagulant and anticoagulant systems [9]. Hyperglycaemia and metabolic disorders are among the significant risk factors of different haemostatic perturbations leading to pro-thrombotic state and cardiovascular severe disorders, including venous thromboembolism, atherosclerosis, myocardial infarction, or stroke [10]. Among different ways to maintain the haemostatic balance and cardiovascular health, a dietary intake of food products rich in polyphenols has been widely suggested [11,12]. Furthermore, multiple functions of phytochemicals on the living cells and their possible synergistic activity contributed to growing interest in applying plant-based foods in the prevention and adjuvant therapy of human diseases [12]. *S. aucuparia* is one of the species of interest due to its wide occurrence, the long tradition of consumption, and several reports on beneficial health properties of rowanberries and extracts derived from them [1,4,5,6,7].

Thus, this study aimed to analyse in vitro the effects of *S. aucuparia* fruit extracts (thoroughly characterised by various phytochemical methods including LC-MS/MS) on some aspects of blood plasma haemostasis and vascular conditions, especially their anticoagulant action and antioxidant protection of blood plasma proteins. Firstly, the effects of the extracts on the peroxynitrite (ONOO^−^)-triggered oxidative/nitrative modifications of human blood plasma fibrinogen were examined, using C-ELISA, SDS-PAGE, and Western blot analyses to evaluate both qualitative and quantitative changes in the fibrinogen structure. The focus on fibrinogen is caused by its important role in maintaining blood fluidity and haemostatic balance. Physiologically, fibrin clot formation from fibrinogen is crucial for stopping bleeding and initiating vascular repair after injury. However, due to the high susceptibility of this protein to different modifications (including oxidative and nitrative damage), fibrinogen functions may be altered and lead to the atypical structure of the fibrin clot and increase its resistance to fibrinolysis [13,14]. The second aspect of our study was the assessment of the anticoagulant potential of the examined extracts, measured by the blood plasma clotting times (APTT, PT, TT) and enzymatic properties of thrombin—an executive serine protease of the blood coagulation cascade, involved not only in haemostatic but also non-haemostatic processes, including the conversion of fibrinogen into fibrin clot and the pro-inflammatory response of endothelial cells [10,15]. The third part of this work aimed to evaluate the inhibitory potential of rowanberry extracts on hyaluronidase activity as an essential modulator of endothelial functions [16]. The experiments on model compounds and correlation studies were used at all study stages to evaluate the contribution of polyphenols to the observed effects.

## 2. Materials and Methods

### 2.1. Plant Material and Extracts Preparation

The analyses were performed using *S. aucuparia* fruit dry methanol-water (1:1, *v*/*v*) extract (ME), and its organic fractions (ME.DEF, diethyl ether fraction; ME.EAF, ethyl acetate fraction; ME.BF, *n*-butanol fraction) obtained as described previously [7]. For extracts preparation, the mature fruits of *S. aucuparia* L. were collected in September 2018 in Lodz, Poland (51°82′ N, 19°55′ E) from ten random plants growing in their natural habitat and separated from each other by several meters to several kilometres. The fruit samples were authenticated by Prof. M.A. Olszewska (Department of Pharmacognosy, Medical University of Lodz, Poland).

The extracts were subjected to thorough phytochemical profiling, the results of which were described earlier [7]. Briefly, the qualitative and quantitative composition was evaluated spectrophotometrically (Folin-Ciocalteu assay, *n*-butanol/HCl assay) and chromatographically (HPLC-PDA, RP/HILIC-UHPLC-PDA-ESI-MS^3^), which enabled to detect 51 various polyphenolic constituents at high levels [7] (Table 1).

### 2.2. Reference Compounds

High-purity standards of model *S. aucuparia* polyphenols, such as chlorogenic acid (5-*O*-caffeoylquinic acid, CHA), isoquercitrin (quercetin 3-*O*-β-D-glucoside, IQ), quercetin 3-*O*-β-sophoroside (SQ), and procyanidin B2 (PB2) were obtained from Sigma-Aldrich (St. Louis, MO, USA) or Phytolab (Vestenbergsgreuth, Germany). In addition, reference compounds used in biological capacity tests, including Trolox (TX) for analyses of the oxidative stress-induced fibrinogen modifications, argatroban (ARG) for testing blood clotting times and enzymatic properties of thrombin, and heparin sodium salt (HP) from porcine intestinal mucosa for hyaluronidase inhibition assay were purchased from Sigma-Aldrich (St. Louis, MO, USA).

### 2.3. Isolation of Human Blood Plasma and Fibrinogen

Human blood plasma was isolated from commercially available buffy coats from healthy volunteers, purchased from the Regional Centre of Blood Donation and Blood Treatment in Lodz (Poland). The isolation was carried out by differential centrifugation, according to Kolodziejczyk-Czepas et al. [17] Fibrinogen was isolated from the obtained plasma by the cold ethanol precipitation, according to Nowak et al. [18] Fibrinogen level in blood plasma was determined using the DiaFib2 Reagent Kit (Diagon, Budapest, Hungary) and attained 3.0–3.7 ± 0.9 g/L. Fibrinogen content in the isolate used for experiments was 2 mg/mL (based on the extinction coefficient: E^1%^ = 15.1 (280 nm)).

The study was carried out following the Declaration of Helsinki, and all experiments were approved by the committee on the Ethics of Research at the University of Lodz (8/KBBN-UŁ/II/2015).

### 2.4. Effects on Oxidative/Nitrative Modifications of Fibrinogen

Fibrinogen or plasma samples were pre-incubated with the examined plant extracts or model compounds (final concentrations: 1–50 μg/mL) for 15 min at room temperature and then treated with ONOO^−^ (100 μM) for 5 min according to Marchelak et al. [19]. ONOO^−^ was synthesized according to Pryor et al. [20]. Samples were diluted with the reducing agent, containing 2-mercaptoethanol (Sigma-Aldrich, St. Louis, MO, USA) and 2× Laemmli Sample Buffer (Bio-Rad, Hercules, CA, USA), and heated at 100 °C for 5 min. Control samples were prepared analogously, using fibrinogen or plasma samples untreated with the analytes or ONOO^−^.

SDS-PAGE and Western blot analyses of the ONOO^−^-induced changes in the isolated fibrinogen and blood plasma fibrinogen, respectively, were performed as described by Marchelak et al. [19]. The molecular weight (MW) on SDS-PAGE and Western Blots was estimated with the Precision Protein Plus Dual Colour standards (Bio-Rad). The densitometric analysis was performed with ImageJ software (National Institutes of Health, Bethesda, MA, USA; Version 1.53e). For details see Supplementary Materials.

The detection of 3-nitrotyrosine-containing fibrinogen (3-NT-Fg) by the competitive enzyme-linked immunosorbent assay (C-ELISA) was performed on fibrinogen samples pre-incubated with the tested extracts or standards (concentration range: 1–50 µg/mL) for 15 min at 37 °C and then treated with ONOO^−^ (100 μM) for 5 min. The C-ELISA assay was carried out according to Khan et al. [21] with modifications described by Kolodziejczyk-Czepas et al. [22]. The standard curve of the nitrated fibrinogen was the basis for calculating the concentration of nitrated proteins that inhibit the binding of the anti-nitrotyrosine antibody. The obtained data were expressed as the 3-NT-Fg equivalents (in nmol/mg of protein) and then presented as a percentage of tyrosine nitration; the content of 3-nitrotyrosine (3-NT) in samples exposed to ONOO^−^ in the absence of the examined extracts and standards was assumed as 100% of tyrosine nitration. The assays were conducted using 96-well plates and a microplate reader SpectrostarNano (BMG Labtech, Ortenburg, Germany).

### 2.5. Effects on Blood Clotting Times and Enzymatic Properties of Thrombin

The activated partial thromboplastin time (APTT), prothrombin time (PT), and thrombin time (TT) tests were determined using blood plasma pre-incubated with the extracts or model compounds (concentration range: 1–50 μg/mL) for 15 min at 37 °C. Control samples were untreated with the analytes. The measurements were carried out using K-3002 optic coagulometer (Kselmed, Grudziądz, Poland) and Diagon (Budapest, Hungary) reagents, based on laboratory protocols delivered by the manufacturer.

Determination of the amidolytic activity of thrombin was performed using thrombin (0.75 U/mL) (Biomed, Lublin, Poland) suspended in 0.05 M Tris-buffered saline (TBS, pH 7.4) and pre-incubated with the examined plant extracts or model compounds (concentration range: 1–50 μg/mL) for 15 min at 37 °C. The analyses employed a kinetic protocol, with absorbance measured at 415 nm, every 10 s, for 15 min. The reaction mixture consisted of thrombin and a 3 mM chromogenic substrate (Chromogenix S-2238; Instrumentation Laboratory, Bedford, MA, USA) in the volume (μL) ratio of 280:40. Thrombin amidolytic activity was estimated based on the maximal velocity of the reaction (V_max_). Control samples (V_max_ = 100%) were prepared using thrombin untreated with the analytes.

Evaluation of the fibrinogen polymerisation (proteolytic activity of thrombin) was performed using the isolated fibrinogen solution in TBS (3 mg/mL) and thrombin (0.75 U/mL, suspended in TBS enriched with 25 mM CaCl_2_; control or pre-incubated with the analytes) in the volume (μL) ratio of 100:200. The measurements were performed at 360 nm, every 10 s, for 20 min, at 37 °C. Effects of the analytes at 1–50 μg/mL were compared to the inhibitory effect of ARG at 0.05–1 μg/mL. The proteolytic activity of thrombin was estimated based on the maximal velocity of the polymerisation process (V_max_). After 60 min, the maximal absorbance (A_max_; λ = 360 nm) of a fully stabilised fibrin clot was measured to indicate the clot density. The assays were conducted using 96-well plates, a microplate reader SpectrostarNano (BMG Labtech, Ortenburg, Germany), and data analysis software (SpectrostarNano Mars, Version 3.01.R2) dedicated to this equipment.

### 2.6. Effects on Hyaluronidase Activity

The ability to inhibit hyaluronidase activity was examined by the turbidimetric method according to Michel et al. [23] with slight modifications. Briefly, the reaction mixture consisted of buffer solutions of the tested analytes, hyaluronidase from bovine testes type I-S (15.0–37.5 U/mL), hyaluronic acid (0.03%, *w*/*v*), and bovine serum albumin (0.1%, *w*/*v*) (Sigma-Aldrich, St. Louis, MO, USA) in the volume (μL) ratio of 20:40:40:250, sequentially added and incubated as previously described [23]. HP was used as a positive control. The results were expressed as IC_50_ values (inhibitory concentration) calculated from the concentration–inhibition curves (5–10 calibration points). For a direct comparison, the results were expressed in equivalents of HP per extract dry weight (mg HP/mg dw).

### 2.7. Statistical Analysis

The results were reported as the means ± SD (standard deviation) or ±SE (standard error) for the indicated number of experiments. The statistical significance of differences between the mean values was determined using a one-way ANOVA (for hyaluronidase activity) or one-way ANOVA for repeated measures (for other experiments), followed by the post hoc Tukey’s test for multiple comparisons or post hoc Dunnett’s test, respectively. All calculations were performed using Satistica13Pl software for Windows (StatSoft, Krakow, Poland) with *p*-values less than 0.05 regarded as significant.

## 3. Results and Discussion

In studies of any plant-based food, the use of properly selected and thoroughly standardised extracts is crucial to obtain reliable results that drive meaningful conclusions on food bioactivity. From the known risk factors of diabetes complications, in this study, we focused on some disturbances of the haemostatic balance, including oxidative modifications of fibrinogen and thrombin activity, as well as hyaluronidase over-activity [13,24]. Therefore, the target extracts were selected according to the results of our previous research [7] that pointed out their significant potential towards human plasma components under oxidative stress conditions. These extracts were: the defatted methanol-water (1:1, *v*/*v*) extract (ME) of rowanberries and its concentrated polyphenol-rich fractions of diethyl ether (ME.DEF), ethyl acetate (ME.EAF), and *n*-butanol (ME.BF), obtained by sequential liquid-liquid partitioning of ME. The extracts were carefully standardised previously by the use of a panel of phytochemical techniques including LC-MS/MS, and the applied analytical approach enabled the detection of 51 phenolics, including pseudodepsides of caffeic and ferulic acids (esters with quinic and shikimic acids), flavonols, proanthocyanidin oligomers and polymers, and simple phenolic acids [7]. The total phenolic content was in the range of 26.03–198.18 mg of gallic acid equivalents (GAE) per g of dry weight, depending on the extract (Table 1). Among the detected compounds, CHA, IQ, SQ, and PB2 were chosen as representatives of different classes of rowanberry phenolics to evaluate the contribution of polyphenols to the tested activities during the present study.

### 3.1. Effects on Oxidative/Nitrative Modifications of Human Fibrinogen

Fibrinogen is a blood plasma glycoprotein that is composed of a pair of three non-identical Aα, Bβ, and γ chains connected with disulphide bonds and form a hexameric structure (340-kDa) [13]. After fibrinopeptides A and B removal via thrombin activity, fibrinogen is converted to fibrin monomers and then polymerised into the fibrin network forming the blood clot [25]. Different modifications of fibrinogen (oxidation, nitration, methylation) may change its clotting ability, the structure of the generated fibrin network, as well as influence the fibrinogen/fibrin interactions with thrombin, endothelial cells, and platelets, leading to thrombosis and other haemostatic disorders [13,25,26]. From various in vivo-generated oxidants and nitrative agents, ONOO^−^ was suggested to be one of the most reactive and linked with diabetes, atherosclerosis, and myocardial infarction [26,27]. Therefore, this oxidant was selected to induce oxidative stress conditions in the present study.

**Figure 1:**
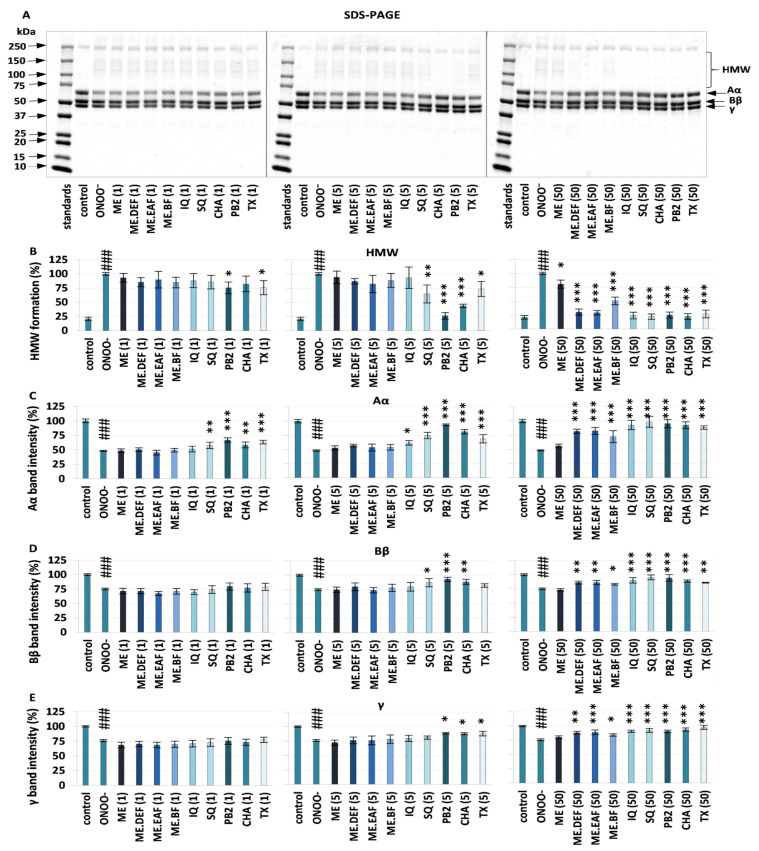
The effects of *S. aucuparia* fruit extracts and their model polyphenols on ONOO^−^-induced changes in the electrophoretic pattern of human fibrinogen: the representative gels of three independent experiments (**A**) (for all gels see Supplementary Materials Figure S2); relative intensities of the bands of the high molecular weight (HMW) aggregates (**B**), the Aα chain bands (**C**), the Bβ chain bands (**D**), and the γ chain bands (**E**) in the fibrinogen samples, calculated based on the densitometric analysis. The protein amount loaded per lane was 10 μg. Results are presented as the means ± SE (*n* = 3). Statistical differences: ### *p* < 0.001 for control fibrinogen versus ONOO^−^-treated fibrinogen (without the analytes); * *p* < 0.05, ** *p* < 0.01, and *** *p* < 0.001 for ONOO^−^-treated fibrinogen in the presence of the analytes (1, 5, 50 µg/mL) versus ONOO^−^-treated fibrinogen in the absence of the analytes. ME, defatted methanol–water extract (1:1, *v*/*v*); ME.DEF, diethyl ether fraction of ME; ME.EAF, ethyl acetate fraction of ME; ME.BF, n-butanol fraction of ME; IQ, isoquercitrin; SQ; quercetin 3-*O*-β-sophoroside; PB2, procyanidin B2; CHA, chlorogenic acid; TX, Trolox.

**Figure 2:**
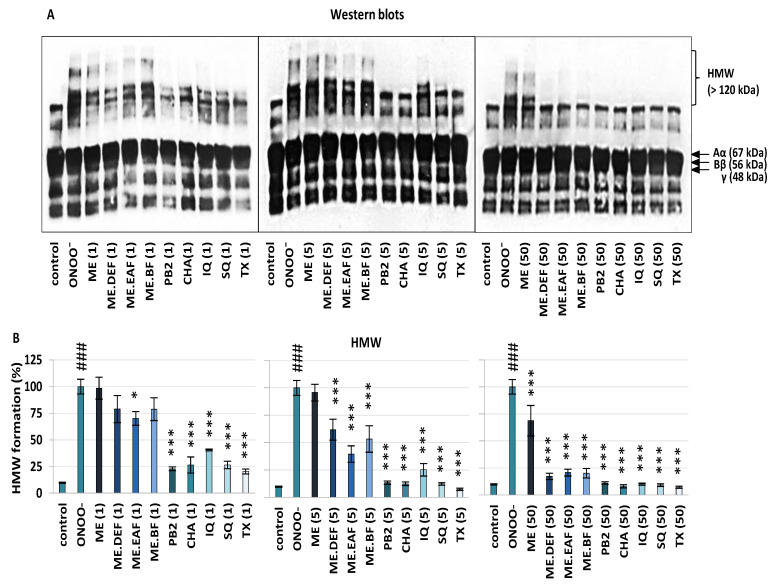
The effects of *S. aucuparia* fruit extracts and their model constituents on ONOO^−^-induced modifications of fibrinogen in blood plasma tested by the Western blot method using the anti-fibrinogen antibody: the representative blots of three independent experiments (**A**) (for all gels see Supplementary Materials Figure S3); and relative intensities of the bands of HMW aggregates, detected in fibrinogen in the plasma matrix (**B**) and calculated based on the densitometric analysis. The protein amount loaded per lane was 8 μg. The bands localised under the γ chain correspond to partial degradation products, occurring under physiological conditions. Results are presented as the means ± SE (*n* = 3). Statistical differences: ### *p* < 0.001 for control sample versus ONOO^−^-treated sample (without the analytes); * *p* < 0.05, and *** *p* < 0.001 for ONOO^−^-treated samples in the presence of the analytes (1, 5, 50 µg/mL) versus ONOO^−^-treated samples in the absence of the analytes. ME, defatted methanol–water extract (1:1, *v*/*v*); ME.DEF, diethyl ether fraction of ME; ME.EAF, ethyl acetate fraction of ME; ME.BF, *n*-butanol fraction of ME; IQ, isoquercitrin; SQ; quercetin 3-*O*-β-sophoroside; PB2, procyanidin B2; CHA, chlorogenic acid; TX, Trolox.

**Figure 3:**
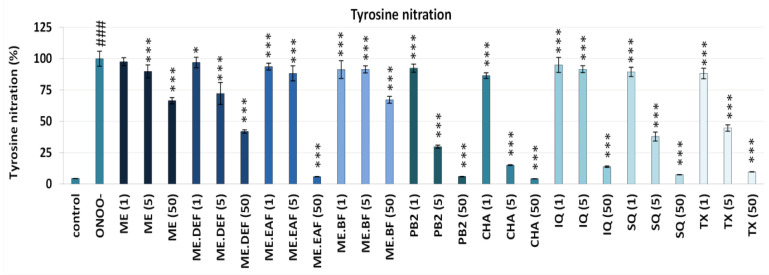
Determination of the protective effects of *S. aucuparia* fruit extracts and their model constituents against the ONOO^−^-induced tyrosine nitration in the isolated human fibrinogen by the C-ELISA. Results are presented as the means ± SE (n = 9). Statistical differences: ### *p* < 0.001 for control fibrinogen versus ONOO^−^-treated fibrinogen (without the analytes); * *p* < 0.05, and *** *p* < 0.001 for ONOO^−^-treated fibrinogen in the presence of the analytes (1, 5, 50 µg/mL) versus ONOO^−^-treated fibrinogen in the absence of the analytes.ME, defatted methanol–water extract (1:1, *v*/*v*); ME.DEF, diethyl ether fraction of ME; ME.EAF, ethyl acetate fraction of ME; ME.BF, *n*-butanol fraction of ME; IQ, isoquercitrin; SQ; quercetin 3-*O*-β-sophoroside; PB2, procyanidin B2; CHA, chlorogenic acid; TX, Trolox.

The ability of the assayed extracts and model polyphenols to protect human fibrinogen against ONOO^−^-generated modifications was evaluated based on SDS-PAGE, Western blot and C-ELISA analyses. The results demonstrated that the presence of the examined plant-derived substances might ameliorate structural changes in the isolated fibrinogen and blood plasma fibrinogen exposed to ONOO^−^, confirmed by both the qualitative and quantitative analyses (Figure 1, Figure 2 and Figure 3). Importantly, there was no pro-oxidative effect noticed in the experiments with the analytes and fibrinogen or plasma but without ONOO^−^.

SDS-PAGE analysis showed that the control fibrinogen samples (without ONOO^−^ and analytes) give an electrophoretic pattern typical for this protein (Figure 1), i.e., three bands corresponding to the molecular weight of 67 kDa, 56 kDa, and 48 kDa (Aα, Bβ, and γ fibrinogen chains, respectively) [18,25]. On the other hand, ONOO^−^-treated samples contained additional bands (MW > 120 kDa), as a result of high-molecular-weight protein aggregates (HMW) generation, as well as a lower band intensity for the Aα, Bβ, and γ fibrinogen chains, among which the Aα chain was the most liable to changes. According to Nowak et al. [18], the highest susceptibility of Aα chains to ONOO^−^ attack might be explained by their exposition on the fibrinogen surface.

As shown in Figure 1, in the presence of the rowanberry extracts, the ONOO^−^-induced fibrinogen modifications were reduced in a dose-dependent manner. At the concentration of 50 µg/mL, the HMW formation was diminished by about 19–71% (Figure 1B), the destruction of the Aα, Bβ and γ chains was significantly prevented—the intensity of the corresponding bands increased (Figure 1C–E), and thereby the electrophoretic pattern of the tested samples was comparable to that of the control fibrinogen (Figure 1A–E), except for the ME-containing samples.

The observed effects were strongly dependent on phenolic composition and correlated (*p* < 0.05) with the total levels of phenolics, flavonoids, mono- and dicaffeoylquinic acids, and other hydroxycinnamic acid derivatives (Table S1). Pure polyphenols CHA and PB2 were the most potent antioxidants in this assay and significantly (*p* < 0.05–0.001) reduced the changes in the fibrinogen structure at the levels as low as 1 µg/mL and 5 µg/mL (Figure 1A–E). The activity of *Sorbus* extracts and model polyphenols was comparable or higher than that of TX—the reference antioxidant.

The protective effect of the tested extracts and compounds on the ONOO^−^-induced alterations of fibrinogen was also confirmed in the blood plasma matrix using the anti-fibrinogen antibody and Western blot analysis (Figure 2).

The results for control plasma (untreated with ONOO^−^ and analytes) showed a common profile of blood plasma fibrinogen, with bands corresponding to Aα_110kDa_, Aα, Bβ and γ chains, as well as the remaining bands (MW < 30 kDa) corresponding to different products of fibrinogen degradation that naturally circulate in the human plasma [18,25]. As in the experiments on the isolated protein, in samples treated with ONOO^−^ only, additional bands of the HMW aggregates were revealed (Figure 2A). In the presence of the extracts and compounds (1–50 µg/mL), the ONOO^−^-triggered HMW formation was reduced by up to 92% (Figure 2B), depending on the analyte and concentration. All tested *Sorbus* polyphenols significantly (*p* < 0.001) reduced modifications of fibrinogen in blood plasma, which was revealed by the reduction of HMW formation by about 59–77%, 75–88%, and 89–92% at 1 µg/mL, 5 µg/mL, and 50 µg/mL, respectively. As for the tested extracts, the strongest activity was found for ME.EAF, with the HMW reduction of approximately 60% at 1 µg/mL (*p* < 0.05) and 79% at 5 µg/mL (*p* < 0.001). However, at 50 µg/mL, all extracts significantly (*p* < 0.001) inhibited the HMW formation, with the lowest activity found for ME.

Apart from the cross-linking of Aα chains that lead to the generation of HMW aggregates, one of the most important structural changes in the fibrinogen molecule caused by the exposure to ONOO^−^ is the transformation of tyrosine into 3-nitrotyrosine (3-NT) units [25,26]. For example, it has been demonstrated that even very low nitration of tyrosine residues in the fibrinogen molecule (i.e., ~45–65 μmol 3-NT/mol of tyrosine) significantly accelerates its polymerisation and may lead to pro-thrombotic effects [28]. Therefore, the activity of the rowanberry extracts against modifications of human fibrinogen was also examined based on the determination of the ONOO^−^-induced 3-NT formation in the isolated fibrinogen by C-ELISA. As expected, a significant (*p* < 0.001) increase in the nitration of tyrosine residues was observed in the fibrinogen samples treated with ONOO^−^, and then in the presence of the extracts and compounds (1–50 µg/mL) the ONOO^−^-triggered tyrosine nitration was considerably inhibited (Figure 3). The reduction in 3-NT formation was dose-dependent and reached up to 96%, depending on the analyte and concentration. The results were statistically significant (*p* < 0.001 or *p* < 0.05) for all cases except ME at 1 μg/mL. Among the extracts, the strongest anti-nitrative activity was revealed for ME.EAF and ME.DEF, with the inhibition in the range of 12–28% and 58–94% at 5 μg/mL and 50 μg/mL, respectively. For pure polyphenols, the highest inhibitory percentage was observed for PB2 and CHA (70–85% at 5 μg/mL and 94–96% at 50 μg/mL), but the anti-nitrative effects of other model compounds, i.e., SQ and IQ, were also remarkable. Moreover, the activity of the extracts and *Sorbus* polyphenols was comparable or higher than that of the positive standard, i.e., TX.

Considering the possible pro-thrombotic consequences of the oxidative/nitrative stress-related changes in the fibrinogen structure, the observed protective effects of *S. aucuparia* fruit extracts towards fibrinogen might form the basis for their functional application and partly explain the use of rowanberries in the traditional treatment of diabetes [1,3] that is often associated with thromboembolic complications [13,24,29,30]. In this context, it is worth noting that the results of this in vitro study were obtained at the levels of rowan polyphenols achievable under physiological conditions. For example, the most active fraction ME.EAF significantly (*p* < 0.05) decreased the tyrosine nitration and formation of HMW aggregates at the levels of 1 µg/mL, 5 µg/mL, and 50 µg/mL (Figure 1, Figure 2 and Figure 3), which corresponds to the total phenolic contents of 0.20 µg GAE/mL, 0.99 µg GAE/mL, 9.91 µg GAE/mL, respectively (the values calculated from Table 1). Bearing in mind the polyphenols bioavailability, these levels may be achieved in plasma after consumption of plant products [31,32,33], and thus the rowanberry extracts might be expected to reduce the negative consequences of oxidative/nitrative alterations of fibrinogen in vivo. As ONOO^−^ generated the fibrinogen modifications, the mechanism behind the observed effects might be due to the direct reaction with ONOO^−^ or other ROS (e.g., ^•^NO_2_, OH^•^, and CO_3_^•−^) formed in the ONOO^−^-induced chain reactions [34]. This agrees with the results of our previous study [7], indicating the high potential of the tested extracts to scavenge multiple oxidants generated in vivo.

So far, only a few plant materials, such as the extract of *Aronia melanocarpa* berries and the *Prunus spinosa* flower extracts, have been tested for their protective activity towards fibrinogen, and different polyphenolic compounds have been indicated as potentially responsible for the observed effects [19,35]. Considering correlation studies (Table S1) and results obtained for model polyphenols (Figure 1, Figure 2 and Figure 3), both hydroxycinnamic acid derivatives and derivatives of flavonols and flavan-3-ols seem to contribute to the target activity of rowanberry extracts. As to the flavan-3-ols, the high activity found for PB2 (Figure 1, Figure 2 and Figure 3), literature data for (−)-epicatechin [36], and lack of correlation between the total proanthocyanidin content and the nitration of tyrosine residues and formation of HMW aggregates (Table S1) suggested that low-molecular-weight derivatives might be more effective protectors of human fibrinogen than high-molecular-weight polymeric proanthocyanidins, prevailing in the rowanberry proanthocyanidin fraction [7]. In turn, in the case of flavonoids, some relationships between their glycosidation degree and the fibrinogen protection might be anticipated. Briefly, SQ—a quercetin diglycoside was a more potent anti-nitrative agent (Figure 3), able to decrease tyrosine nitration by about 62% at concentration 5 µg/mL, than a quercetin monoglycoside—IQ. Nevertheless, both quercetin derivatives significantly (*p* < 0.05) reduced the negative consequences of oxidative/nitrative alterations of fibrinogen, which is in agreement with the literature data that indicate the importance of *ortho*-dihydroxyl structure for the protective activity of flavonoids towards fibrinogen [19].

### 3.2. Effects on Blood Clotting Times, Enzymatic Properties of Thrombin, and Fibrin Density

The influence on the haemostatic activity of blood plasma was evaluated based on well-known diagnostic parameters, i.e., blood clotting times, such as APTT, PT, and TT. At the tested concentrations (1–50 μg/mL), the examined rowanberry extracts did not affect the APTT and PT (*p >* 0.05) (Figure S1), though slightly higher APTT values for CHA and SQ at 1 μg/mL and 50 μg/mL, respectively, as well as a PT value for PB2 at 50 μg/mL, were observed. In samples treated with a reference anticoagulant drug (ARG) at 0.5–5 μg/mL, the APTT and PT were considerably prolonged (168–396% and 132–541% of control plasma, respectively). On the other hand, slightly prolonged TT (*p <* 0.05), recorded for plasma samples treated with ME.DEF and ME.BF at 50 μg/mL (about 2.3%), as well as PB2 (about 3%), suggested that the examined plant extracts might act as thrombin inhibitors (Figure S1). Moreover, it was previously reported that fruit extracts of other *Sorbus* species (*S. commixta*) might also inhibit thrombin activity (e.g., TT was prolonged by about 47% at the concentration of 2.5 mg/mL of ethylacetate fraction) [37]. Therefore, the next step of the present study was evaluating the influence of the rowanberry fruit extracts and compounds on thrombin activity.

**Figure 4:**
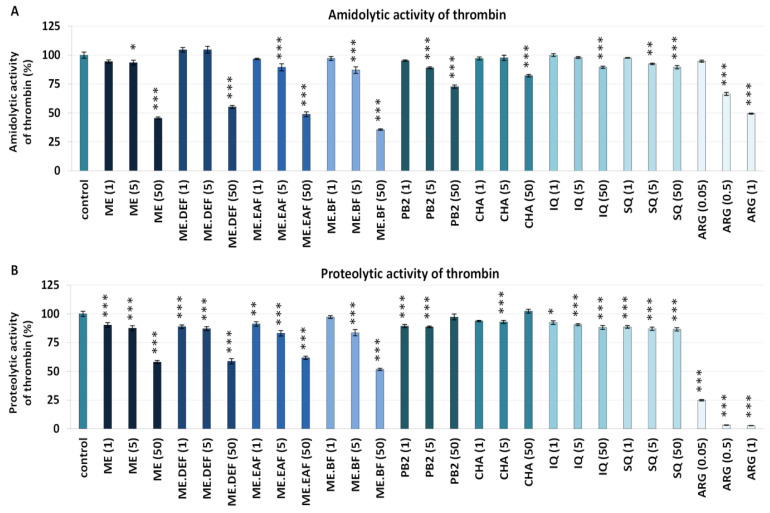
Inhibitory effects of the *S. aucuparia* fruit extracts and their model constituents on the amidolytic (**A**) and proteolytic (**B**) activity of thrombin. Both assays were performed using a kinetic protocol and calculated based on the V_max_ parameter. Results are presented as the means ± SE (*n* = 9). Statistical differences: * *p* < 0.05, ** *p* < 0.01, and *** *p* < 0.001 for samples in the presence of the analytes (1, 5, 50 µg/mL) or the reference inhibitor, argatroban (ARG, 0.05, 0.5, 1 µg/mL) versus control samples. ME, defatted methanol–water extract (1:1, *v*/*v*); ME.DEF, diethyl ether fraction of ME; ME.EAF, ethyl acetate fraction of ME; ME.BF, *n*-butanol fraction of ME; IQ, isoquercitrin; SQ; quercetin 3-*O*-β-sophoroside; PB2, procyanidin B2; CHA, chlorogenic acid.

As shown in Figure 4A, the extracts inhibited the amidolytic activity of thrombin in a concentration-dependent manner with statistically significant effects (*p <* 0.05) observed at 5 μg/mL or 50 μg/mL, depending on the extract. Analyses of the V_max_ showed that their inhibitory potential reached up to 64% for ME.BF at 50 μg/mL, and was only slightly lower for ME, ME.DEF and ME.EAF (45–55% at 50 μg/mL). In comparison, the V_max_ value for ARG at 1 μg/mL was about 50%. The inhibitory activity of the tested model compounds was less evident and reached up to 27% for PB2 at 50 μg/mL (Figure 4A). However, significant (*p* < 0.05) correlations between the percentage inhibition of thrombin activity and phenolic levels were noticed (Table S1), which might suggest some synergistic effects between individual polyphenols.

**Figure 5:**
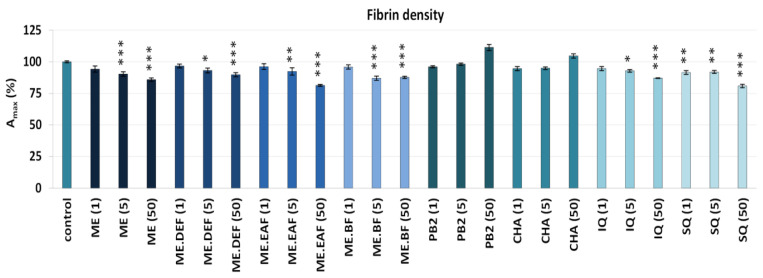
Effects of the *S. aucuparia* fruit extracts and their model polyphenols on the fibrin stabilisation phase in the fibrin(ogen) polymerisation tests. The fibrin density was determined as the maximum absorbance (A_max_), and the A_max_ values of control samples were assumed as 100%. Results are presented as the means ± SE (*n* = 9). Statistical differences: * *p* < 0.05, ** *p* < 0.01, and *** *p* < 0.001 for samples in the presence of the analytes (1, 5, 50 µg/mL) versus control samples. ME, defatted methanol–water extract (1:1, *v*/*v*); ME.DEF, diethyl ether fraction of ME; ME.EAF, ethyl acetate fraction of ME; ME.BF, *n*-butanol fraction of ME; IQ, isoquercitrin; SQ; quercetin 3-*O*-β-sophoroside; PB2, procyanidin B2; CHA, chlorogenic acid.

The ability to inhibit thrombin activity was also examined based on the fibrinogen proteolysis (proteolytic activity of thrombin) (Figure 4B). All tested extracts effectively reduced the rate of fibrinogen polymerisation, which was shown as a significant (*p* < 0.05) decrease of the V_max_ parameter. Similar to the case of amidolytic activity, the effectiveness of rowanberry extracts on fibrinogen proteolysis was superior compared to that of model polyphenols—for example, the V_max_ values in the presence of the extracts at 50 μg/mL decreased by about 38–48%, while in the presence of polyphenols by only 13% at most. Moreover, in samples treated with the extracts at 5 μg/mL and 50 μg/mL, a slight decrease in the A_max_ parameter was revealed (Figure 5), indicating a lower density of the generated fibrin. As for the pure polyphenols, only IQ and SQ influenced the stabilisation of the fibrin clot.

The thrombin-catalysed proteolytic conversion of fibrinogen to fibrin is the critical step in both extrinsic and intrinsic coagulation pathways, and enhanced generation of this enzyme is associated with numerous disorders related to the cardiovascular system [9,10]. For example, increased thrombin activity noted under hyperglycaemia or oxidative stress conditions was suggested to be responsible for enhanced fibrin generation and clot density [10,30]. For that reason, thrombin has become one of the most critical targets for anti-thrombotic therapies and prevention of the thromboembolic complications of diseases [38]. In this context, even a slight prolongation of the TT (Figure S1) and significant (*p* < 0.05) inhibition of the enzymatic activity of thrombin (Figure 4) might contribute to the health benefits of rowanberries. Notably, the thrombin-inhibitory effect of the examined extracts was observed not only based on the hydrolysis of a synthetic substrate (i.e., H-d-phenylalanyl-l-pipecolyl-l-arginine-*p*-nitroaniline dihydrochloride) that is often used for in vitro studies but also during the proteolysis of fibrinogen, a physiological substrate for this enzyme [38].

The ability to inhibit the thrombin proteolytic activity was previously reported, e.g., for the polyphenol-rich chokeberry and grape seeds extracts [39], the bufadienolide-rich *Kalanchoe daigremontiana* root fraction [40], and the alkaloid and phenolic-rich *Uncaria tomentosa* leaf and bark extracts [15]. Moreover, different types of plant constituents have been investigated as potential thrombin inhibitors, including various types of polyphenols [41], and the relationship between the structure of the compounds and their activity has been examined [15,42,43]. Based on molecular docking studies, it has been established that polyphenols might be either direct or indirect thrombin inhibitors [15]. As it is known, four structural components of thrombin are mainly responsible for its enzymatic properties, including the active site with the catalytic triad of His_57_, Asp_102_, and Ser_195_ residues, the anion-binding exosites 1 and 2, and the Na^+^ binding site [38]. While compounds such as caffeic acid, CHA, quercetin and (−)-epicatechin have been reported to join directly to the active site, procyjanidins B2 and C1 have also been able to bound near the exosite 1, responsible for thrombin-fibrinogen interactions, and exosite 2 that is known to bind heparin and platelet trans-membrane glycoproteins [15]. Moreover, different molecules might have lower or higher affinity to thrombin, based on the set of thrombin residues they interact with and types of interactions, including the number of hydrogen bonds, as well as electrostatic and van der Waals interactions [42,43].

Considering the literature data [15,42,43] and the present study results (Figure 4, Figure S1, Table S1), the capacity of the rowanberry extracts to inhibit thrombin activity might be partly attributed to the presence of flavan-3-ol derivatives, flavonols, and hydroxycinnamic acid derivatives. However, although the antithrombin potential of different polyphenols was confirmed (*p* < 0.05), their activity was considerably lower than that of the extracts (Figure 4). This agrees with previous findings, which suggested that most of the phenolic compounds in a pure form are relatively weak or moderate thrombin inhibitors [42,43]. Therefore, the observed rowanberry effects may partly be due to the synergy between individual compounds in direct or indirect interactions [15]. On the other hand, the significant correlation (*p* < 0.05) between the total content of oligomeric and polymeric proanthocyanidins and thrombin activity (Table S1) suggests that high-molecular-weight proanthocyanidins might be potent thrombin inhibitors. Indeed, according to previous findings [15], the affinity to thrombin increases with the polymerisation degree, i.e., flavan-3-ol monomer < dimer < trimer. Thus, it may be the reason for the tremendous inhibitory potential found for ME and ME.BF, which contained high levels of proanthocyanidins (Table 1) with prevailing polymers [7].

Additionally, it is worth noticing that the tested extracts not only inhibited the enzymatic properties of thrombin but also decreased the density of the generated fibrin (Figure 5). Since the fibre density reduction makes the structure more permeable to proteolytic enzymes and enhances the fibrinolytic system efficiency [44], this effect might also contribute to the anti-thrombotic potential of rowanberries. According to the results obtained for model polyphenols, flavonoids might be mainly responsible for the influence of the extracts on the fibrin properties. Indeed, quercetin and IQ were previously reported to significantly reduce fibrin density and clotting process [45]. Moreover, some reports point out that other phenolics, such as (−)-epicatechin, make the fibrin clot structure less dense and more permeable [46].

### 3.3. Effects on Hyaluronidase Activity

The blood coagulation and fibrinolytic systems are tightly regulated, and endothelial cells are integral in the haemostatic balance. Under physiological conditions, endothelial cells are activated only after a vessel injury to promote thrombin generation and modulate the haemostatic response. However, under pathological conditions, dysfunctional endothelium may trigger the activation of blood platelets and plasma coagulation proteins, leading to the formation of undesirable fibrin clots [16]. Indeed, degradation of the endothelial glycocalyx in consequence of hyaluronidase activity or ROS-induced oxidation was reported as one of the variables responsible for the increased risk of thrombosis and the development of vascular complications of diabetes [29,47]. Therefore, the present study evaluated the influence of the rowanberry extracts on hyaluronidase activity.

As shown in Table 2, the concentration-dependent ability to inhibit the enzyme activity was revealed for all extracts. The most substantial effect was observed for ME.DEF with activity only slightly lower than that of HP (a known hyaluronidase, thrombin and coagulation factor Xa inhibitor) [48]. However, the activity of the extracts turned out to be only slightly related to the phenolic content (*r* = 0.7599, *p* = 0.24; Table S1). Indeed, in the tested concentration range, the inhibitory potential of model polyphenols was lower than 50% (IC_50_ values not reached).

The ability of rowanberry extracts to inhibit hyaluronidase (Table 2) might be behind the number of possible health-promoting outcomes and one of the mechanisms suggesting the use of rowanberries as a functional food in the prevention of civilisation diseases. Natural hyaluronidase inhibitors have been investigated for decades as potent regulating agents, and polyphenolic compounds are among the best recognized [49]. For instance, it was established that *ortho*-dihydroxyl structure and a double bond between carbon atoms C2 and C3 of the flavonoid skeleton increased their inhibitory potential, and thus quercetin derivatives were proven to be among the most effective hyaluronidase inhibitors [50]. Moreover, it was suggested that flavonols might be both competitive and non-competitive hyaluronidase inhibitors with the ability to bind to the active site of the enzyme [50,51]. On the other hand, flavan-3-ols like (+)-catechin and (−)-epicatechin were assumed to interact with different, so-called catechin-binding sites, and modulate enzyme activity by changing its conformation [52]. Furthermore, the synergistic activity of various polyphenols should also be considered because it might explain the relatively moderate inhibitory potential of pure polyphenols, observed in this and other studies [50,53] in comparison to whole plant extracts. However, since there is no previous research on the synergy effects of individual polyphenols towards hyaluronidase, future studies should address this issue directly.

## 4. Conclusions

The present study broadens the current state of knowledge on the biological activity of rowan fruits by new data regarding their influence on plasma haemostasis and vascular conditions in vitro. The results revealed that rowanberry extracts could protect human fibrinogen against oxidative modifications, inhibit the enzymatic properties of thrombin, decrease the density of the generated fibrin clot, as well as inhibit the hyaluronidase activity. These mechanisms might contribute to the anti-thrombotic effect and the protective impact on the endothelial functions. Thus, the obtained results may partly confirm the health benefits of rowanberries as food and explain their traditional application in cardiovascular complications of diabetes. Moreover, the experiments on model compounds and correlation studies revealed the contribution of different groups of polyphenols and their possible synergistic activity to the observed effects. Thus, the presented results indicate that rowan fruits are a valuable source of biologically active compounds and promising plant material for functional applications and encourage further studies on other haemostasis-related parameters, including platelets activity and potential anti-inflammatory effects. In particular, in vivo studies are desirable to verify the expected fruit impact in humans.

## Figures and Tables

**Table 1:** Content of the primary polyphenols in the tested extracts (mg/g dry weight) according to Rutkowska et al. [7].

	ME	ME.DEF	ME.EAF	ME.BF
Total phenolic content	26.03 ± 0.33	155.96 ± 2.99	198.18 ± 8.53	76.93 ± 1.78
Total content of mono- and dicaffeoylqunic acids	8.70 ± 0.11	103.09 ± 1.12	187.99 ± 5.50	41.78 ± 0.61
3-*O*-caffeoylquinic acid	2.93 ± 0.04	11.25 ± 0.08	21.64 ± 0.54	14.51 ± 0.22
5-*O*-caffeoylquinic acid (CHA)	4.88 ± 0.04	35.12 ± 0.15	140.18 ± 3.40	23.84 ± 0.35
4-*O*-caffeoylquinic acid	0.36 ± 0.01	3.65 ± 0.02	8.10 ± 0.23	1.88 ± 0.03
1-*O*-caffeoylquinic acid	0.20 ± 0.00	6.01 ± 0.22	6.81 ± 0.12	1.55 ± 0.02
3,5-*O*-dicaffeoylquinic acid	0.33 ± 0.01	32.05 ± 0.17	10.38 ± 0.31	n.d.
1,3-*O*-dicaffeoylquinic acid	n.d.	10.93 ± 0.45	n.d.	n.d.
4,5-*O*-dicaffeoylquinic acid	n.d.	4.07 ± 0.03	0.89 ± 0.02	n.d.
Total content of other hydroxycinnamic acid derivatives	1.05 ± 0.02	33.52 ± 1.05	51.75 ± 0.66	21.10 ± 0.30
Total content of hydroxybenzoic acid derivatives	n.d.	2.64 ± 0.04	n.d.	n.d.
Total content of flavonoids	0.62 ± 0.00	1.43 ± 0.03	5.79 ± 0.14	5.76 ± 0.06
quercetin 3-*O*-β-sophoroside (SQ)	0.39 ± 0.00	n.d.	n.d	3.57 ± 0.04
quercetin 3-*O*-β-D-galactoside	0.03 ± 0.00	n.d.	1.36 ± 0.03	0.38 ± 0.01
quercetin 3-*O*-β-D-glucoside (IQ)	0.07 ± 0.00	0.37 ± 0.01	2.25 ± 0.05	0.28 ± 0.00
quercetin	n.d.	1.06 ± 0.01	0.34 ± 0.02	n.d.
Total content of oligomeric and polymeric proanthocyanidins	14.18 ± 0.37	n.d.	n.d.	13.34 ± 0.46

Results are presented as the means ± SD (*n* = 3). The total phenolic content was determined by Folin-Ciocalteu assay in gallic acid equivalents (GAE); the total content of polymeric proanthocyanidins was determined by *n*-butanol/HCl assay in cyanidin chloride equivalents (CyE); the content of other compounds and groups of compounds was determined by RP-HPLC-PDA in equivalents of the authentic standards. ME, defatted methanol–water extract (1:1, *v*/*v*); ME.DEF, diethyl ether fraction of ME; ME.EAF, ethyl acetate fraction of ME; ME.BF, *n*-butanol fraction of ME. Additional abbreviation: n.d., not detected.

**Table 2:** Inhibitory activity of *S. aucuparia* fruit extracts on hyaluronidase.

Analyte	IC_50_ (µg/mL) ^a^	IC_50_ (mg HP/ mg dw) ^b^
ME	151.54 ± 8.99 E	0.32 ± 0.02
ME.DEF	87.05 ± 4.55 B	0.56 ± 0.03
ME.EAF	108.53 ± 3.95 C	0.45 ± 0.02
ME.BF	118.79 ± 2.45 D	0.41 ± 0.01
PB2	>120	-
CHA	>120	-
IQ	>200	-
SQ	>200	-
HP	48.98 ± 3.71 A	1.00

Results are presented as the means ± SD (*n* = 3) calculated per dry weight of the extract or standard (heparin sodium salt, HP). Different superscripts (capitals) indicate significant differences in the means at *p* < 0.05. Inhibitory concentrations (amount of an analyte needed for 50% inhibition of the enzyme activity) are expressed as follows: ^a^ in µg of the dry extract or standard/ mL of the enzyme solution; ^b^ in mg of the HP/mg of the dry extract or standard (based on HP activity). ME, defatted methanol–water extract (1:1, *v*/*v*); ME.DEF, diethyl ether fraction of ME; ME.EAF, ethyl acetate fraction of ME; ME.BF, *n*-butanol fraction of ME; IQ, isoquercitrin; SQ; quercetin 3-*O*-β-sophoroside; PB2, procyanidin B2; CHA, chlorogenic acid.

## Data Availability

Data is contained within the article.

## Supplementary Materials

The following are available online at https://www.mdpi.com/article/10.3390/antiox10122009/s1, Materials and Methods: Effects on oxidative/nitrative modifications of fibrinogen; Table S1: Correlation coefficients (*r*) and probability (*p*) values of the linear relationships between phenolic contents of S. aucuparia fruit extracts and their activity parameters; Figure S1: The effects of *S. aucuparia* fruit extracts and their model constituents on blood clotting times of human plasma: activated partial thromboplastin time, APTT (A); prothrombin time, PT (B); and thrombin time, TT (C); Figure S2: Raw images of all electrophoresis gels (SDS-PAGE). For detail information see Manuscript Figure 1; Figure S3. Raw images of all Western blots. For detail information see Manuscript Figure 2.
