# Supplementary material for: The Effects of Sorbus aucuparia L. Fruit Extracts on Oxidative/Nitrative Modifications of Human Fibrinogen, Impact on Enzymatic Properties of Thrombin, and Hyaluronidase Activity In Vitro
Source: Antioxidants (Basel). 2021 Dec 18;10(12):2009. doi: 10.3390/antiox10122009 (PMC8698768; doi:10.3390/antiox10122009)
Supplement: Supplementary file 1 [file antioxidants-10-02009-s001.zip › antioxidants-1482329-supplementary.pdf]

Supplementary materials:

The Effects of *Sorbus aucuparia* L. Fruit Extracts on Oxidative/Nitrative Modifications of Human Fibrinogen, Impact on Enzymatic Properties of Thrombin, and Hyaluronidase Activity In Vitro

Magdalena Rutkowska[1,*] Joanna Kolodziejczyk-Czepas[2] and Monika Anna Olszewska[1]

[1] Department of Pharmacognosy, Faculty of Pharmacy, Medical University of Lodz, 1 Muszynskiego St.,90-151 Lodz, Poland; monika.olszewska@umed.lodz.pl
[2] Department of General Biochemistry, Faculty of Biology and Environmental Protection, University of Lodz, 141/143 Pomorska St., 90-236 Lodz, Poland; joanna.kolodziejczyk@biol.uni.lodz.pl
* Correspondence: magdalena.rutkowska@umed.lodz.pl

Materials and Methods

Effects on Oxidative/Nitrative Modifications of Fibrinogen

SDS-PAGE analysis of the $ONOO^-$-induced changes in the isolated fibrinogen was performed according to Marchelak et al.[19] using a Mini-Protean Electrophoresis Cell (Bio-Rad) and 4-20% Mini-PROTEAN TGX Precast Gels (Bio-Rad). The running buffer was composed of 10x Tris/Glycine/SDS running buffer (Bio-Rad, Hercules, CA, USA) and deionized water (1:9, *v*/*v*). The protein was stained using a mixture of 0.125% (*w*/*v*) Coomassie Brillant Blue R250 (Thermo Scientific, Waltham, MA USA), 10% (*v*/*v*) acetic acid, 50% (*v*/*v*) methanol, and deionised water. Then, the Coomassie dye was removed by aspiration, and gels were covered with the destaining solution (10% acetic acid, 25% methanol, deionized water), which was changed several times until the background staining was removed.

Western-blot analysis of the $ONOO^-$-induced changes in the blood plasma fibrinogen was performed using 4-15% Mini-PROTEAN TGX Precast Gels (Bio-Rad) as described above for SDS-PAGE analysis, but without the staining and destaining steps [19]. The electrophoretic transfer onto Immobilon®-P polyvinylidene fluoride (PVDF) membrane (Sigma-Aldrich) was performed using the Mini Trans-Blot Cell (Bio-Rad). The membrane was activated by wetting in methanol for 5 seconds and then rinsing with deionised water. The transfer buffer was a mixture of 25 mM Tris, 192 mM glycine, 20% (*v*/*v*) methanol, 0.05% (*w*/*v*) SDS and deionized water (pH 8.3). The overnight transfer in a Mini Trans-Blot Module (Bio-Rad) was carried out at 4°C, 30 V. The membrane was blocked for 1.5 h with 5% defatted dry milk solutions in TBS-T, and incubated for 1 h with the 1:5000 dilution of Goat Anti-Fibrinogen Antibody (Sigma-Aldrich). The membrane was washed six times for 5 min each with TBS-T, incubated for 1 h with the 1:5000 dilution of Rabbit Anti-Goat IgG H&L (HRP) Secondary Antibody (Abcam, Cambridge, UK), and then washed again. The bands corresponding to fibrinogen were visualised by luminol-enhanced chemiluminescence (ECL) system and recorded on the X-ray film.

The molecular weight (MW) on SDS-PAGE and Western Blots was estimated with the Precision Protein Plus Dual Colour standards (Bio-Rad). The MW of an unknown band was determined using a standard curve of the log (MW) versus the retention factors R_f of the standards. The strong linear relationship ($r > 0.99$, $p < 0.05$) between the protein MW and migration distances confirmed the reliability in predicting MW. Densitometric analysis was performed with ImageJ software (National Institutes of Health, Bethesda, MA, USA; Version 1.53e).

Results

Table S1. Correlation coefficients (*r*) and probability (*p*) values of the linear relationships between phenolic contents of *S. aucuparia* fruit extracts and their activity parameters.

r(p) for:	**Total phenolics**	**Total mono- and dicaffeoylquinic acids**	**Total other hydroxycinnamic acid derivatives**	**Total hydroxybenzoic acid derivatives**	**Total flavonoids**	**Total oligomeric and polymeric proanthocyanidins**
**SDS-PAGE HMW**	**0.9728 (0.000)***	**0.9097 (0.000)***	**0.9587 (0.000)***	0.5716 (0.052)	**0.7900 (0.002)***	0.1321 (0.682)
**SDS-PAGE Aα**	**0.9580 (0.000)***	**0.8896 (0.000)***	**0.9420 (0.000)***	0.5607 (0.058)	**0.7915 (0.002)***	0.1735 (0.590)
**SDS-PAGE Bβ**	**0.8402 (0.001)***	**0.7746 (0.003)***	**0.8352 (0.001)***	0.5089 (0.091)	**0.7187 (0.008)***	0.1198 (0.711)
**SDS-PAGE γ**	**0.8776 (0.000)***	**0.8027 (0.002)***	**0.8479 (0.000)***	0.4788 (0.115)	**0.7481 (0.005)***	0.3331 (0.290)
**Western blot HMW**	**0.7962 (0.002)***	**0.7261 (0.008)***	**0.7970 (0.002)***	0.4495 (0.143)	**0.7406 (0.006)***	0.1857 (0.563)
**Tyrosine nitration**	**0.9538 (0.000)***	**0.9517 (0.000)***	**0.9395 (0.000)***	0.3830 (0.219)	**0.7403 (0.006)***	0.1126 (0.728)
**APTT**	0.0042 (0.990)	-0.0095 (0.977)	0.0486 (0.881)	0.0662 (0.835)	0.1009 (0.755)	-0.1991 (0.535)
**PT**	-0.3547 (0.258)	-0.3379 (0.283)	-0.3624 (0.247)	-0.0013 (0.997)	-0.4545 (0.138)	-0.3008 (0.342)
**TT**	**0.6034 (0.038)***	0.5345 (0.073)	**0.6188 (0.032)***	0.4015 (0.196)	**0.5952 (0.041)***	0.0671 (0.836)
**Amidolytic activity**	**0.7098 (0.010)***	**0.6101 (0.035)***	**0.6761 (0.016)***	0.2650 (0.405)	**0.7786 (0.003)***	**0.6999 (0.011)***
**Proteolytic activity**	**0.7267 (0.007)***	**0.6109 (0.035)***	**0.6830 (0.014)***	0.3669 (0.241)	**0.7424 (0.006)***	**0.6674 (0.018)***
**Fibrin density**	**0.6721 (0.017)***	**0.6701 (0.017)***	**0.6650 (0.018)***	0.0471 (0.884)	**0.6943 (0.012)***	0.4280 (0.165)
**Hyaluronidase activity**	0.7599 (0.240)	0.5809 (0.419)	0.6785 (0.321)	0.8375 (0.162)	0.0609 (0.939)	-0.8275 (0.172)

Activity and concentration parameters according to Fig. 1–5, S1 and Table 1–2. For correlation studies, the activity parameters were calculated as the percentage of the increase (SDS-PAGE bands intensity for Aα, Bβ, and γ chains; and APTT, PT, and TT) or the decrease (SDS-PAGE bands intensity for HMW aggregates; Western blot HMW intensity; tyrosine nitration; amidolytic and proteolytic activity of thrombin; and fibrin density), depending on the desired effect on the tested parameter in the samples treated with the investigated extracts versus the $ONOO^-$-treated plasma in the absence of the analytes (for SDS-PAGE, Western blot and C-ELISA assays) or control samples (for blood clotting times and thrombin activity tests). The influence on hyaluronidase activity was expressed as equivalents of HP per extract dry weight (mg HP/mg dw). Asterisks mean statistical significance of the estimated linear relationships (*$p < 0.05$). All statistically significant correlations are printed in bold.

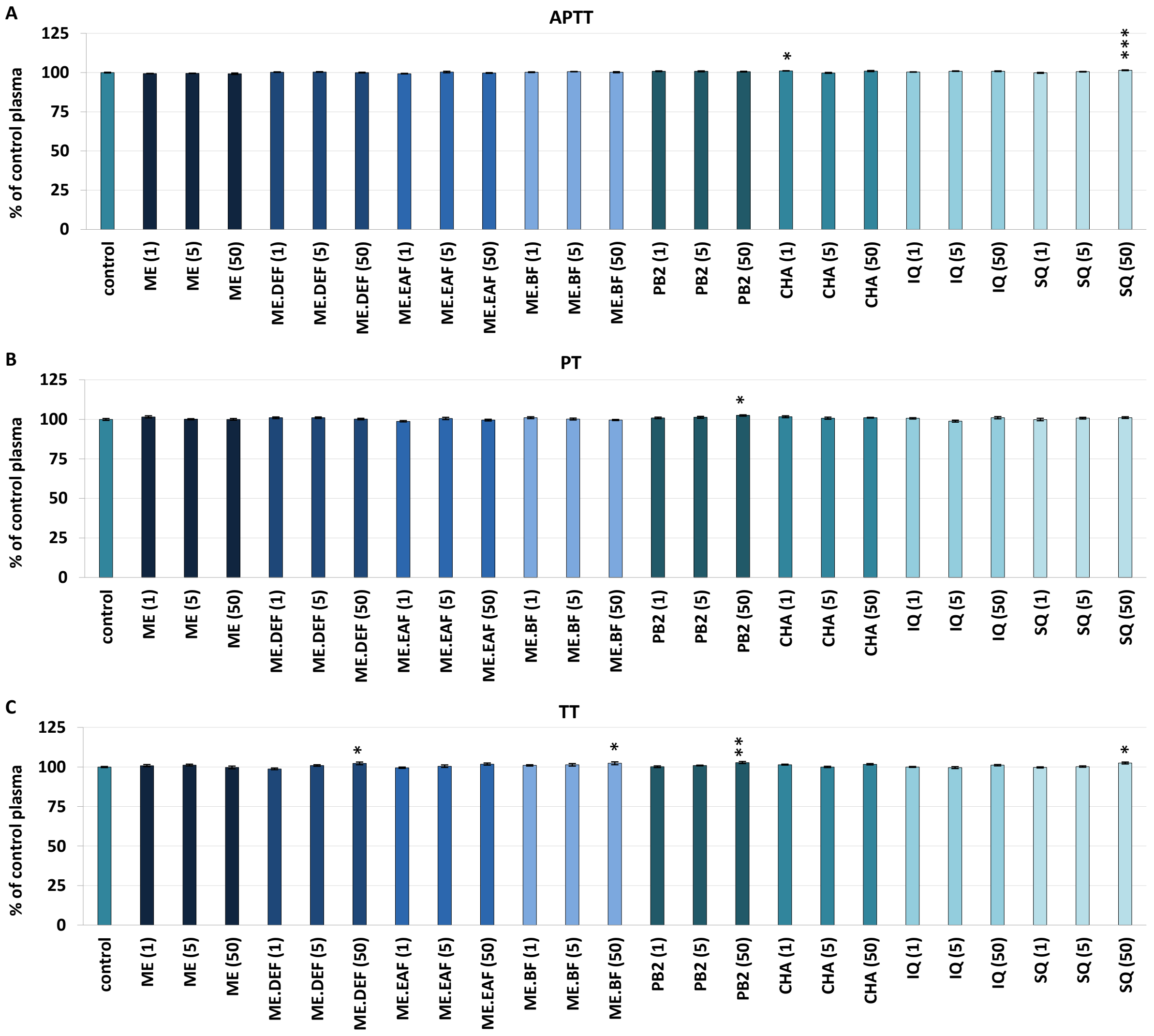

Figure S1. The effects of *S. aucuparia* fruit extracts and their model constituents on blood clotting times of human plasma: activated partial thromboplastin time, APTT (A); prothrombin time, PT (B); and thrombin time, TT (C). The clotting times were monitored in seconds (s) and calculated as % of the control plasma time assumed as 100%. Results are presented as means ± SE ($n = 10$). Statistical differences:* $p < 0.05$, ** $p < 0.01$, and *** $p < 0.001$ for samples in the presence of the analytes (1, 5, 50 µg/mL) versus control plasma. ME, defatted methanol–water extract (1:1, *v*/*v*); ME.DEF, diethyl ether fraction of ME; ME.EAF, ethyl acetate fraction of ME; ME.BF, *n*-butanol fraction of ME; IQ, isoquercitrin; SQ; quercetin 3-*O*-β-sophoroside; PB2, procyanidin B2; CHA, chlorogenic acid. The clotting times for the reference compound, argatroban (ARG, 0.5 µg/mL) were as follow: APTT, 77.27 ± 7.17 s (168% of the value for control plasma); PT, 20.93 ±1.43 s (132% of control plasma); TT, 140.71 ± 12.88 s (831% of control plasma).

Experiment 1

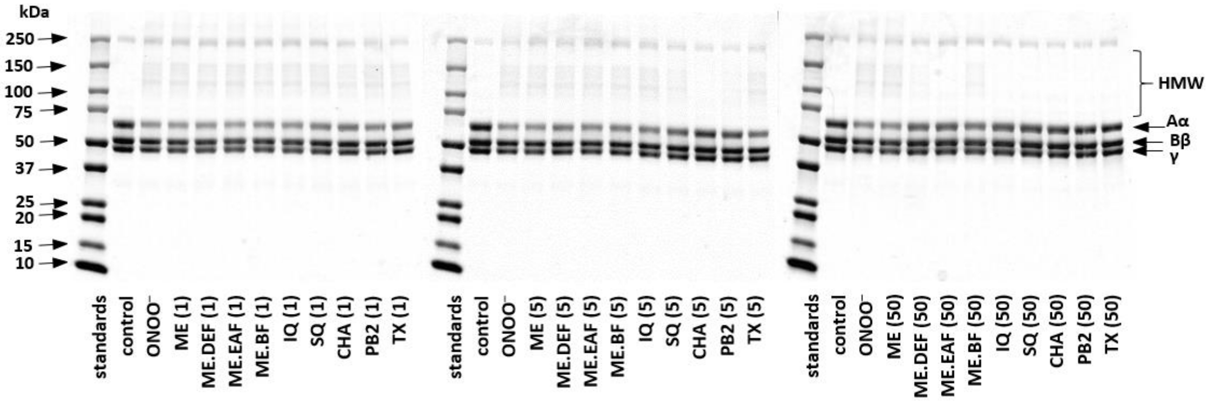

Experiment 2

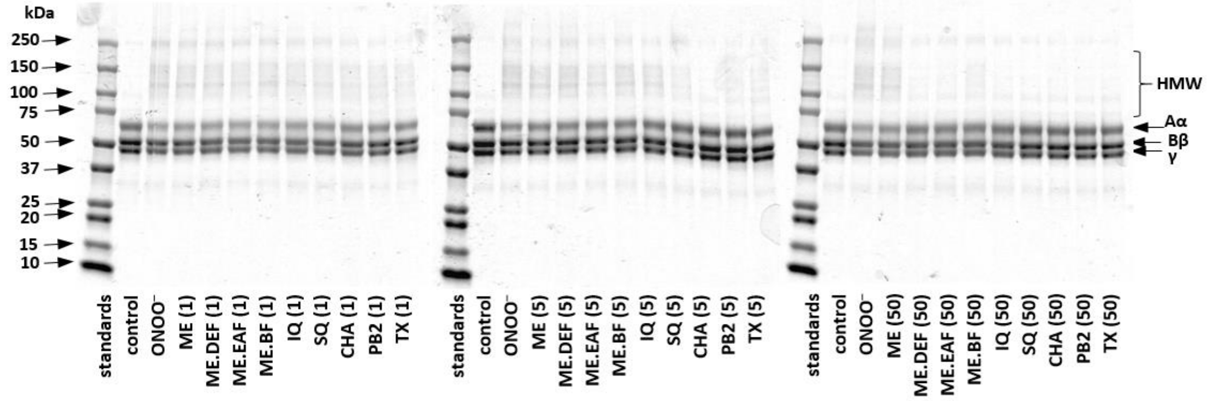

Experiment 3

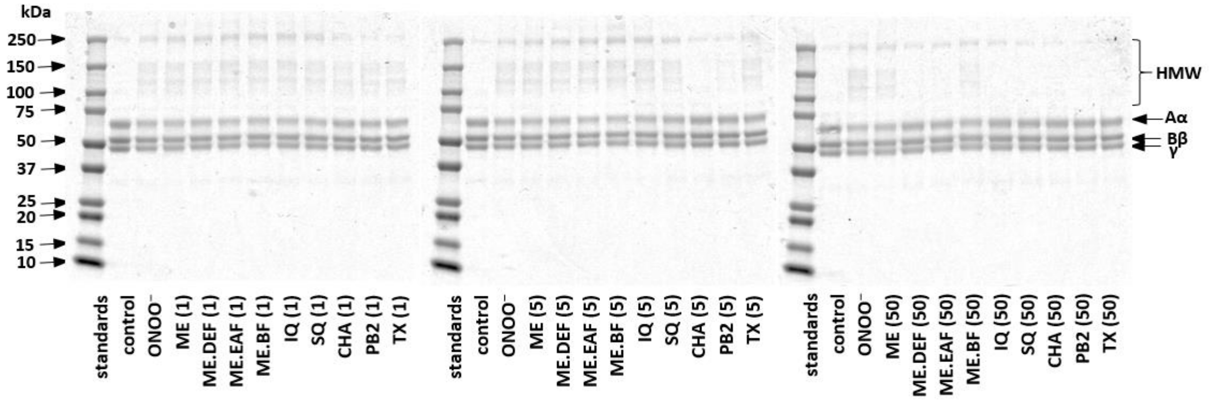

Figure S2. Raw images of all electrophoresis gels (SDS-PAGE). For detail information see Manuscript Figure 1.

Experiment 1

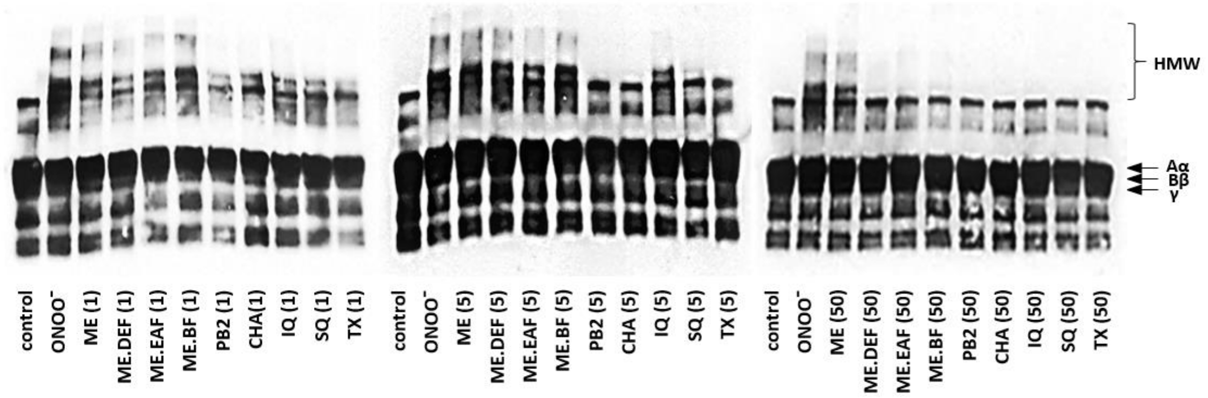

Experiment 2

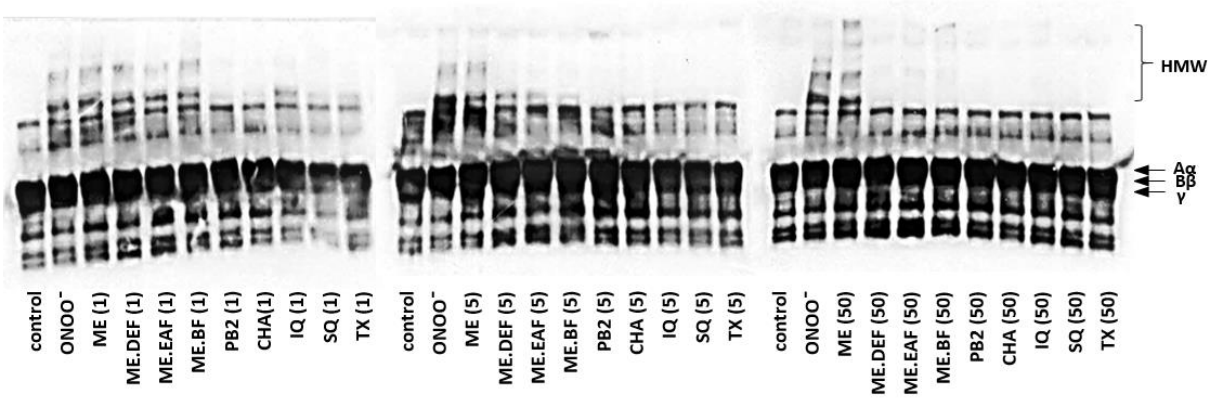

Experiment 3

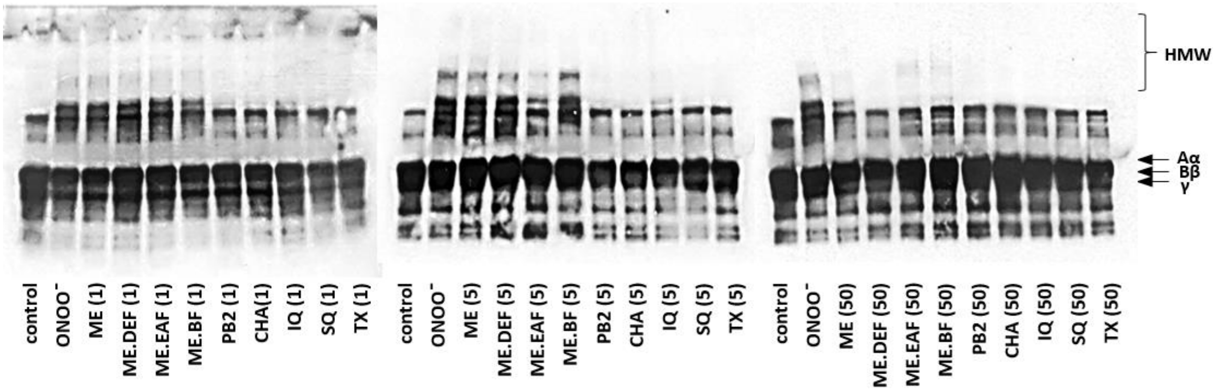

Figure S3. Raw images of all Western blots. For detail information see Manuscript Figure 2.
